# Effectiveness of single dose oral dexamethasone versus multidose prednisolone for treatment of acute exacerbations of asthma among children

**DOI:** 10.1016/j.amsu.2022.104799

**Published:** 2022-11-08

**Authors:** Asma Tayyab, Alvina Asif, Shaista Qazi, Sughra Wahid, Anam Zafar, Michael Halim, Irshad Hussain, Hassan Mumtaz

**Affiliations:** a Senior Registrar Paediatrics, Islamabad Medical and Dental College, Pakistan; b Head of Department Paediatrics, KRL Hospital Islamabad, Pakistan; c Senior Medical Officer KRL Hospital Islamabad, Fazaia Medical College, Pakistan; d University of Salford, Greater Manchester, United Kingdom; e Registrar Paediatrics, KRL Hospital Islamabad, Pakistan; f Clinical Research Associate, Maroof International Hospital, Islamabad, Public Health Scholar: Health Services Academy, Islamabad, Pakistan

**Keywords:** Dexamethasone, Prednisolone, Acute exacerbation, Asthma, Requirement of systemic steroids

## Abstract

**Introduction:**

Asthma is one of the most common chronic diseases in children and worldwide its prevalence has increased dramatically in the last three decades. We aimed to compare single dose oral dexamethasone versus multiple doses of oral prednisolone in children with acute exacerbation of asthma in terms of post treatment requirement of systemic steroids.

**Materials and methods:**

This Randomized control trial has been conducted in the Department of paediatrics, KRL Hospital, Islamabad from Dec 2018 to June 2019.312 patients between the age of 2–12 years patients were randomized into Group A receiving a STAT single dose of oral dexamethasone 0.3 mg/kg and Group B receiving prednisolone 1 mg/kg/day followed by two doses on Day 2 and 3. further dose of systemic steroids were ascertained through PRAM score.

**Results:**

In this study mean age in Group A was 8 years with SD ± 5.68 while mean age in Group B was 7 years with SD ± 6.12. In Group A 58% patients were male and 42% patients were female. Whereas in Group B 59% patients were male and 41% patients were female. In Group A 12% patients had further requirement of systemic steroids while in Group B 18% patients had further requirement of systemic steroids while 82% patients didn't had further requirement of systemic steroids.

**Conclusion:**

Our study concludes that post treatment requirement of systemic steroids is less in single dose oral dexamethasone as compare to multiple doses of oral prednisolone in children with acute exacerbation of asthma.

## Highlights

• This trial compares single dose oral dexamethasone versus multiple doses of oral prednisolone in asthmatic children.
• Post treatment requirement of systemic steroids is less in single dose oral dexamethasone.

## 1 Introduction

Asthma is the most common chronic condition in children and the main cause of visits to the emergency room [1]. An estimated 300 million individuals around the world have asthma, according to the WHO [2]. According to GINA (Global Initiative for Asthma) [3,] the prevalence of asthma is 4–5% in Pakistan and 9.1% in Bangladesh.

Inflammation in the lungs over time can lead to asthma, which causes the airways to enlarge, restrict, and react excessively [4]. According to the World Health Organization, "uncontrolled asthma that can result in danger of frequent severe exacerbations (or death) and/or adverse reactions to therapies and/or persistent morbidity" [5] is considered severe asthma.

In cases of moderate to severe asthma exacerbations, systemic corticosteroids are required [1]. By reducing airway inflammation, these drugs aid in the immediate management of asthma symptoms and also reduce the frequency with which bronchodilators are required. One typical steroid regimen consists of 5 days of oral prednisolone [4]. The available data, however, suggest that patients are not consistently compliant with this regimen, and that it has low palatability [1]. 64% of children in one study took their oral corticosteroid treatment for the full duration. The long duration of prednisolone treatment and the possibility of adverse effects, such as unpleasant taste and nausea and vomiting, may reduce patient compliance [6].

An additional drug that has been studied is dexamethasone. It is more efficient and has a longer half-life (36–72 h versus 12–36 h for prednisolone), therefore less of it is required. Dexamethasone offers the advantages of being easier to take and requiring fewer unscheduled check-ins [1]. Those who have used it to treat bacterial meningitis and croup in children report no ill effects. In order to ensure compliance without the more invasive intramuscular injection, a single oral dose of dexamethasone could be administered [6].

Numerous randomised controlled trials have been reported comparing the use of dexamethasone and prednisolone to treat acute asthma exacerbations in children [6]. Recent studies [1] show that dexamethasone, when given for a shorter length of time, is as efficacious as prednisone in treating mild to severe asthma exacerbations presented to the emergency room. It was found by Greenberg et al. that there was no statistically significant difference between the relapse rates of those on dexamethasone (16%) and those taking prednisolone (8%) [7]. Qureshi et al. [8] found that the relapse rates for dexamethasone were 7.4% and for prednisolone they were 6.9%.

Asthma is a common health problem that affects many children in the Pakistan. Patients receive nebulized 2 agonists and anticholinergics in addition to oral and IV corticosteroids. However, no studies have been conducted on our population to distinguish between the two drugs. The results of this study on acute exacerbation of asthma may lead to improved patient compliance and fewer relapses in the emergency care environment. In this study, we will evaluate the efficacy of oral dexamethasone against prednisolone in treating acute exacerbations of asthma in children.

## 2 Materials And Methods

This Randomized control trial has been conducted at KRL Hospital, Islamabad from Dec 2018 to June 2019. A Sample size of 312 was calculated by using WHO formula with Confidence level = 95%, Study power = 80%, Level of significance = 5%. Anticipated proportion of prednisolone was 6.9% [8] ^&^ Anticipated proportion of dexamethasone was 16% [7]. Non-probability consecutive sampling technique was used.

Our study is fully compliant with the CONSORT 2010 guidelines [9]. A complete STROCSS 2021 checklist has been provided as a supplementary file. UIN researchregistry8288 [10] identifies our study in Research Registry. Our research adheres to the principles outlined in the Helsinki Declaration.

### 2.1 Inclusion & exclusion criteria

Patient having Age 2–12years of Both gender having Previous history of asthma as diagnosed by a physician and those Presenting with acute exacerbation of asthma with symptoms of cough, wheeze, dyspnoea and oxygen saturation of less than 95% and with PRAM score of more than or equal to 6 were included in our study.

Patients with severe or life-threatening asthma, such as those with a quiet chest, cyanosis, extreme fatigue, or an inability to communicate verbally. Those who had been previously diagnosed with tuberculosis, had a temperature above 39.5° Celsius, had taken corticosteroids within the previous four weeks, or had a significant comorbid lung, cardiac, immune, liver, endocrine, neurological, or psychiatric disease were not included in our study.

## 3 Data collection

The KRL Hospital ethics board approved the data collection. The parents of the children participating in the trial provided written consent after being informed of the study's goals. The data was collected using a form of the Paediatric Respiratory Assessment Measure (PRAM) that has been updated and proven reliable for use with children. All data will be collected and documented by the investigator herself.

Every single participant was randomly assigned to one of two treatment groups. Patients in Group A received an oral dexamethasone 0.3 mg/kg (maximum 12 mg) dosage STAT in the ED, while patients in Group B received prednisolone 1 mg/kg/day (maximum 40 mg) for three days straight (Days 2, 3, and 4). Parents have been asked to contact the emergency room immediately if they see any worsening of their child's symptoms. The child was thoroughly evaluated at the follow-up appointment, and the necessity for additional systemic steroid treatment was determined using the PRAM score.

### 3.1 Analysis of data

SPSS version 21 was used to enter and analyse the data. The average and standard deviation for quantitative factors including age and PRAM score at presentation, discharge, and subsequent visit were calculated. Quantitative measures were used for quantitative variables including gender and the need for further systemic steroids during the follow up visit, whereas qualitative variables were measured using frequency and percentage. The chi-square test was used to compare the need for further systemic steroids between the two treatment groups, and a P value of less than 0.05 was regarded to indicate statistical significance. Stratification was used to account for potential confounding factors, such as age, gender, and pre-test PRAM score. In this study, we used a chi-square test after stratifying the samples and considered a P value .05 to indicate statistical significance.

## 4 Results

In this study age distribution was analysed as in Group A 59(38%) patients were in age range 2–6 years and 97(62%) patients were in age range 7–12 years. Mean age was 8 years with SD ± 5.68. Whereas in Group B 56(36%) patients were in age range 2–6 years and 100(64%) patients were in age range 7–12 years. Mean age was 7 years with SD ± 6.12. Gender distribution was analysed as in Group A 90(58%) patients were male and 66(42%) patients were female. Whereas in Group B 92(59%) patients were male and 64(41%) patients were female, as shown in Table 1.

**Table 1:** Patient demographics.

AGE	GROUP A	GROUP B	P-value
**2**–**6 years**	59(38%)	56(36%)	0.13
**7**–**12 years**	97(62%)	100(64%)	
**Gender**			
**Male**	90(58%)	92(59%)	0.81
**Female**	66(42%)	64(41%)	

Baseline PRAM score was analysed as in Group A 23(15%) patients had PRAM score ≤6 and 133(85%) patients had PRAM score >6. Mean PRAM score was 8 with SD ± 2.83. Whereas in Group B 20(13%) patients had PRAM score ≤6 and 136(87%) patients had PRAM score >6. Mean PRAM score was 8 with SD ± 2.94.

PRAM score at discharge was analysed as in Group A 122(78%) patients had PRAM score ≤6 and 34(22%) patients had PRAM score >6. Mean PRAM score was 5 with SD ± 2.86. Whereas in Group B 100(64%) patients had PRAM score ≤6 and 56(36%) patients had PRAM score >6. Mean PRAM score was 6 with SD ± 3.12.

PRAM score at follow up was analysed as in Group A 137(88%) patients had PRAM score ≤6 and 19(12%) patients had PRAM score >6. Mean PRAM score was 3 with SD ± 2.01. Whereas in Group B 128(82%) patients had PRAM score ≤6 and 28(18%) patients had PRAM score >6. Mean PRAM score was 5 with SD ± 2.87as shown in Table 2.

**Table 2:** PRAM SCORE at baseline, discharge & follow-up.

PRAM SCORE	GROUP A	GROUP B	P-value
BASELINE PRAM SCORE			
**< 6**	23(15%)	20(13%)	1.00
**>6**	133(85%)	136(87%)	
**PRAM SCORE AT DISCHARGE**			
**< 6**	122(78%)	100(64%)	0.00
**>6**	34(22%)	56(36%)	
**FOLLOW UP**			
**< 6**	137(88%)	128(82%)	0.00
**>6**	19(12%)	28(18%)	

Further requirement of systemic steroids was analysed as in Group A 19(12%) patients had further requirement of systemic steroids while 137(88%) patients had no further requirement of systemic steroids. Whereas Group B 28(18%) patients had further requirement of systemic steroids while 128(82%) patients had no further requirement of systemic steroids.

Stratification of Further requirement of systemic steroids with respect to age, gender and baseline PRAM score is shown in Table 3.

**Table 3:** Stratification of systemic steroids with respect to age, gender and baseline PRAM score.

AGE	SYSTEMIC STEROIDS	GROUP A	GROUP B	P value
Age				
**2**–**6 years**	Yes	7	10	0.3654
	No	52	46	
**Total**		59	56	
**7**–**12 years**	Yes	12	18	0.2716
	No	85	82	
**Total**		97	100	
**Gender**				
**Male**	Yes	11	17	0.2421
	No	79	75	
**Total**		90	92	
**Female**	Yes	8	11	0.4136
	No	58	53	
**Total**		66	64	
**BASELINE PRAM SCORE**				
**< 6**	Yes	3	4	0.5376
	No	20	16	
**Total**		23	20	
**>6**	Yes	16	24	0.1954
	No	117	112	
**Total**		133	136	

## 5 Discussion

Our study shows that mean age in Group A was 8 years with SD ± 5.68 while mean age in Group B was 7 years with SD ± 6.12. In Group A 58% patients were male and 42% patients were female. Whereas in Group B 59% patients were male and 41% patients were female. In Group A 12% patients had further requirement of systemic steroids while in Group B 18% patients had further requirement of systemic steroids while 82% patients had no further requirement of systemic steroids.

Similarly, Paniagua N et al. [11] found no difference in asthma symptoms and quality of life at day 7 between oral doses of dexamethasone (0.6 mg/kg/dose, maximum dose 12 mg) and a five-day course of prednisone/prednisolone (1.5 mg/kg/day, maximum 60 mg, on day 1 and 1 mg/kg/day, maximum 60 mg, on days 2 through 5).

16 children in the dexamethasone group got additional systemic steroids (13.1%) within 14 days following trial enrolment, compared to 5 children in the prednisolone group (4.2%) (absolute difference 8.9%; 95% CI 1.9%–16.0%) (Cronin JJ et al. [12]).

In a study published in 2001, Qureshi et al. [13] compared the effects of dexamethasone (0.6 mg/kg daily; maximum 16 mg/d) for two days to those of prednisone for five days. Hospitalization (11% vs 12%, P = .84), recurrence (7.4% vs 6.9%, P = .84), and symptom persistence at 10 days (22% vs 21%) were all comparable with the other treatment. The drug's recipients had better compliance and less negative side effects, like nausea and vomiting.

When comparing dexamethasone (0.6 mg/kg daily) to a higher dose of prednisone (2 mg/kg daily), no statistically significant differences were identified in recurrence rates (16% vs 8%, P = .27) or in the incidence of vomiting (10% vs 18%, P = .24) in paediatric patients with asthma exacerbations [14]. However, the study's limitations can be attributed to its small sample size and a mid-study shift in hospital procedure. For children aged 2–16 years with mild to moderate asthma, a single dose of dexamethasone revealed no difference in hospital admission rates or time to return to baseline of patient self-assessment scores, as investigated by Altamimi et al. [15].

Asthma exacerbations of all severity levels, from mild to severe, can benefit from short courses of systemic corticosteroids. Both prednisone (1–2 mg/kg daily for 5 days) and dexamethasone (0.3–0.6 mg/kg daily for 1–5 days) are reasonable options. More research is required to determine the best systemic steroid dose, duration, and selection [16].

## 6 Conclusion

Our study concludes that post treatment requirement of systemic steroids is less in single dose oral dexamethasone as compare to multiple doses of oral prednisolone in children with acute exacerbation of asthma.

## Ethical approval

Ethical approval was obtained by KRL Hospital, ref no ERC-17/02/17.

## Please state any sources of funding for your research

Nill.

## Author contribution

Author Contribution:

1. Conceptualization: Asma Tayyab.

2. Formal Analysis: Alvina Asif, Shaista Qazi.

3. Writing – review and editing: Hassan Mumtaz, Michael Halim.

4. Writing –original draft:, Irshad Hussain, Sughra Wahid, Anum Zafar,

## Registration of research studies

1. Name of the registry: Research registry.

2. Unique Identifying number or registration ID: researchregistry8288.

Browse the Registry - Research Registry.

## Guarantor

Hassan Mumtaz, Irshad Hussain.

## Conent

Studies on patients or volunteers require ethics committee approval and fully informed written consent which should be documented in the paper.

Authors must obtain written and signed consent to publish a case report from the patient (or, where applicable, the patient's guardian or next of kin) prior to submission. We ask Authors to confirm as part of the submission process that such consent has been obtained, and the manuscript must include a statement to this effect in a consent section at the end of the manuscript, as follows: "Written informed consent was obtained from the patient for publication of this case report and accompanying images. A copy of the written consent is available for review by the Editor-in-Chief of this journal on request”.

Patients have a right to privacy. Patients’ and volunteers' names, initials, or hospital numbers should not be used. Images of patients or volunteers should not be used unless the information is essential for scientific purposes and explicit permission has been given as part of the consent. If such consent is made subject to any conditions, the Editor in Chief must be made aware of all such conditions.

Even where consent has been given, identifying details should be omitted if they are not essential. If identifying characteristics are altered to protect anonymity, such as in genetic pedigrees, authors should provide assurance that alterations do not distort scientific meaning and editors should so note.

## Provenance and peer review

Not commissioned, externally peer-reviewed.

## Data collection

The informed consent from the patients was obtained considering Helsinki's Declaration.

## Contribution statement

All authors have equally contributed to the manuscript and have approved the final manuscript to be published.

## Declaration of competing interest

No conflict of interests declared by the authors.
